# EXAMINING THE COST BURDEN OF DIETARY SUPPLEMENTS IN OLDER ADULT DRIVERS: AN ANALYSIS FROM THE AAA LONGROAD STUDY

**DOI:** 10.1093/geroni/igae098.2450

**Published:** 2024-12-31

**Authors:** Sara Baird, Ryan Moran, Sarah Hacker, Dylan Lawton, Linda Hill

**Affiliations:** University of California San Diego, La Jolla, California, United States; University of California San Diego, La Jolla, California, United States; University of California San Diego, La Jolla, California, United States; University of Hawaii, Honolulu, Hawaii, United States; University of California San Diego, La Jolla, California, United States

## Abstract

Dietary supplements (DS) are commonly used, yet consistent evidence of benefit remains lacking. Many older adults have fixed incomes, and the relative costs of DS remain poorly understood. Data was obtained from the first two years from the LongROAD study, a multicenter prospective cohort study of older drivers (≥ 65 years old). At baseline and annually, over-the-counter and prescription medications were systematically coded and categorized, with attention to DS formulations. Cost estimates utilizing the lowest priced brand on Amazon.com were matched to registered DS in the National Institutes of Health Office of Dietary Supplements database. Cost burden per participant was based on reported frequency of use and analysed against participant demographics. Of the 2,990 participants at baseline, 2717 participants had follow up through year 2. Almost all participants (n=2518, 84%) utilized at least one DS during the two-year period. Of 146 DS formulations identified, 135 (92%) were included for cost analysis; the remainder were not listed on Amazon.com. Among DS users, the average estimated annual cost burden was $205.15 (SD +/- $259.49) at baseline, $222.35 (SD +/- $282.68) at year one, and $213.56 (SD +/- $273.51) at year two. Female participants had a higher average estimated annual cost (p< 0.001); there was no significant difference by education or income level. The average monthly cost of DS in this population is similar to two or more generic medications. The health implications and potential cost effect on prescription medication compliance warrants further attention.

